# Neuromodulation for treatment-resistant depression: Functional network targets contributing to antidepressive outcomes

**DOI:** 10.3389/fnhum.2023.1125074

**Published:** 2023-03-02

**Authors:** Shaquia L. Idlett-Ali, Claudia A. Salazar, Marcus S. Bell, E. Baron Short, Nathan C. Rowland

**Affiliations:** ^1^Department of Neurosurgery, University of Colorado Anschutz Medical Campus, Aurora, CO, United States; ^2^Department of Neurosurgery, Medical University of South Carolina, Charleston, SC, United States; ^3^Department of Psychiatry and Behavioral Sciences, Medical University of South Carolina, Charleston, SC, United States

**Keywords:** deep brain stimulation, epidural cortical stimulation, neuromodulation, transcranial magnetic stimulation (repetitive), salience network, treatment-resistant depression, electroconvulsive therapy

## Abstract

Non-invasive brain stimulation is designed to target accessible brain regions that underlie many psychiatric disorders. One such method, transcranial magnetic stimulation (TMS), is commonly used in patients with treatment-resistant depression (TRD). However, for non-responders, the choice of an alternative therapy is unclear and often decided empirically without detailed knowledge of precise circuit dysfunction. This is also true of invasive therapies, such as deep brain stimulation (DBS), in which responses in TRD patients are linked to circuit activity that varies in each individual. If the functional networks affected by these approaches were better understood, a theoretical basis for selection of interventions could be developed to guide psychiatric treatment pathways. The mechanistic understanding of TMS is that it promotes long-term potentiation of cortical targets, such as dorsolateral prefrontal cortex (DLPFC), which are attenuated in depression. DLPFC is highly interconnected with other networks related to mood and cognition, thus TMS likely alters activity remote from DLPFC, such as in the central executive, salience and default mode networks. When deeper structures such as subcallosal cingulate cortex (SCC) are targeted using DBS for TRD, response efficacy has depended on proximity to white matter pathways that similarly engage emotion regulation and reward. Many have begun to question whether these networks, targeted by different modalities, overlap or are, in fact, the same. A major goal of current functional and structural imaging in patients with TRD is to elucidate neuromodulatory effects on the aforementioned networks so that treatment of intractable psychiatric conditions may become more predictable and targeted using the optimal technique with fewer iterations. Here, we describe several therapeutic approaches to TRD and review clinical studies of functional imaging and tractography that identify the diverse loci of modulation. We discuss differentiating factors associated with responders and non-responders to these stimulation modalities, with a focus on mechanisms of action for non-invasive and intracranial stimulation modalities. We advance the hypothesis that non-invasive and invasive neuromodulation approaches for TRD are likely impacting shared networks and critical nodes important for alleviating symptoms associated with this disorder. We close by describing a therapeutic framework that leverages personalized connectome-guided target identification for a stepwise neuromodulation paradigm.

## Introduction

Treatment-resistant depression (TRD) is defined as major depressive disorder (MDD) unresponsive to one or more conscripted treatments (Souery et al., 2006). Estimated failure rates of MDD treatment are as high as 10–30%, however, the type and number of treatments as well as the length of administration required for TRD diagnosis are inconsistently defined in the literature (Al-Harbi, 2012). Only 30% of TRD patients ever achieve remission using other means, and another 30% attempt suicide at least once in their lifetime (Bergfeld et al., 2018; Cole et al., 2022).

Measuring connectivity of brain networks for psychiatric disorders has become an increasingly used analytical technique, because it has the potential to illuminate neuromodulation targets for both non-invasive, e.g., TMS and invasive, e.g., DBS, approaches (Hollunder et al., 2022). This is especially critical since both modalities have considerable drawbacks. TMS for TRD requires up to 6 weeks of daily treatment sessions, and DBS may not show consistent efficacy in some patients until a year or more after implantation. Thus, an algorithm that helps clinicians identify and define TRD subtypes and suggests patient-specific treatments is needed.

Though many neural networks exhibit dysfunction in the setting of TRD, a close reading of the psychiatric literature consistently correlates default mode, salience, and central executive network dysfunction with TRD presentation and may suggest etiology (Hamilton et al., 2013). In contrast, a close reading of the neurosurgical literature for TRD highlights cortico-basal ganglia limbic circuits as well as cognitive control, reward and other networks that might be targeted based on specific symptom domains, including affect and attention (Williams et al., 2021). Compounding the problem of separate taxonomies in the literature is the fact that there is not enough crosstalk between the two disciplines, threatening to extend the semantic confusion surrounding these topics. Fortunately, the overall impression from examining these two bodies of literature is that the structures and networks described are very similar, if not the same, being targeted using these different approaches. As an example, functional connectivity studies show that DLPFC and subcallosal cingulate cortex (SCC), the two most common targets of TMS and DBS, respectively, are co-activated during effective treatment of TRD patients (Anderson et al., 2016). The implication is that a convergence of network anatomy and connectivity relationships among various neuromodulation treatments for TRD are needed to highlight similarities between them and advance care of TRD patients in a meaningful way.

In this review, we discuss the evidence accumulated in favor of pursuing a neuromodulatory approach for TRD. Putative mechanisms for both TMS and DBS are reviewed as well as an alternative procedure, epidural cortical stimulation, that likely engages similar circuitry. We conclude with a synthesis of network topology and argue that this may form the basis for developing a stepwise interventional approach to TRD, increasing in invasiveness only when a less invasive approach fails. In summary, much more work is needed to define clinical outcomes associated with a network-centric approach to treating TRD, however, this paper lays the important groundwork for consolidating the concepts and terminology for future *trans*-disciplinary discourse and action.

## Neurobiology of transcranial magnetic stimulation

TMS is a non-invasive modality that has been FDA-approved in the United States for the management of treatment-resistant depression, smoking cessation and obsessive compulsive disorder, and is being investigated for numerous other psychiatric disorders such as addiction, chronic pain, anxiety, panic, and post-traumatic stress disorder (Cohen et al., 2022). Delivery of therapy is accomplished using a pulsatile electromagnetic field induced in a coil held at the surface of the scalp that stimulates underlying cortex (Barker et al., 1985). Electrophysiological simulations and patch-clamp studies suggest TMS-induced action potentials occur at or near somata rather than the axon hillock, the axon itself, or dendrites (Pashut et al., 2011). Computational modeling has shown that fibers running parallel to the surface of the scalp are more likely to be stimulated compared to oblique fibers, and vertical fibers are most resistant to stimulation (Tofts, 1990). Somatic depolarization drives both orthodromic and antidromic action potential propagation. Orthodromic propagation causes action potentials and activity-dependent plasticity in an anterograde manner. Antidromic propagation promotes dendritic spine growth of stimulated neurons through a local Ca^2+^ spike, leading to increased presynaptic connectivity (Pashut et al., 2011). White matter architecture changes also occur over time via synaptic strengthening and pruning (Anderson et al., 2016).

## Evolution of clinical experience with TMS in treatment-resistant depression

George et al. (2000) were the first to publish results of a double-blind, sham-controlled study showing the antidepressant potential of TMS over left DLPFC in randomized subjects (George et al., 2000). TMS had been introduced years earlier by Pascual-Leone for motor cortical stimulation (Pascual-Leone et al., 1994). In the George study, 30 medication-free patients with major depressive and bipolar disorder were treated with TMS daily for 2 weeks. Twenty patients were assigned to the active TMS group, whereas 10 were assigned to the sham group. TMS sessions lasted for 20 min and occurred each weekday. All patients underwent cerebral blood flow single photon emission computed tomography (SPECT) at the start (1 day), middle (5 day), and 3 days after the end of the study. The authors concluded that daily TMS over left DLPFC produced a significant antidepressant response, defined as greater than 50% improvement in baseline Hamilton Rating Scale for Depression (HRSD) scores. In the same year, Berman et al. also assessed the efficacy of TMS in unmedicated TRD patients using a randomized, double-blind design. TRD patients enrolled in the trial were assigned to either active TMS (*N* = 10) or sham (*N* = 10) treatment. The active TMS group received 20 2 s trains of 20 Hz with 58 s intervals daily over a 2 week course (Berman et al., 2000). As in George’s study, results showed statistically significant reductions in depressive symptoms compared to the sham group. These two important papers instigated a groundswell of interest in TMS for depression and many other psychiatric disorders.

Today, TMS is practiced globally, and TRD remains the primary indication. The procedure is well tolerated; individuals who receive TMS report a > 50% decrease in symptom severity (Lisanby et al., 2009; George et al., 2010; O’Reardon et al., 2010). Despite this, one obstacle that has remained in scaling its deployment even further is the burdensome requirement for multiple treatments. For example, an initial treatment regimen consists of 40–60 min sessions of active treatment, 5 days per week for 3–6 weeks (Holtzheimer et al., 2010). Because of this, innumerable attempts have been made in the two decades since George’s study to modify the TMS protocol with the goal of shortening the amount of therapy needed. In 2018, the US Food and Drug Administration (FDA) approved intermittent theta-burst stimulation (iTBS) as a new variant of TMS for the treatment of TRD (Mendlowitz et al., 2019). FDA approval was contingent on several new studies of theta-burst stimulation, including a multi-center clinical trial by Blumberger et al. (2018) that compared the efficacy of iTBS to conventional TMS in patients with TRD (Blumberger et al., 2018). In their study, TRD patients were randomized to iTBS (*N* = 209) or 10 Hz TMS (*N* = 205). Patients were treated with the modality they were randomized to for 5 days a week for 4–6 weeks. The 10 Hz TMS sessions lasted 37 min and consisted of 3,000 pulses per session. The iTBS session consisted of triplet 50 Hz pulses repeated at 5 Hz for 600 pulses over only 3 min. The HRSD was administered after each of the sessions and 1-, 4-, and 12 weeks after treatment. They observed that scores significantly improved in both iTBS and TMS groups (overall reduction in HRSD-17 scores was 10.1 points in the iTBS group and 9.9 points in the 10 Hz TMS group) at baseline and 1 week after treatment. This trial became the formative study establishing iTBS as a safe, tolerable, and effective treatment for people with TRD. Furthermore, iTBS sessions last only a few min and are less costly than conventional TMS.

Cole and colleagues addressed another well-known challenge with TMS protocols: overall treatment duration. In the SAINT study, patients with TRD were enrolled (*N* = 22) and received 5 days of iTBS (Cole et al., 2020). Each day consisted of ten daily sessions, for a total of 18,000 pulses per day. Functional MRI identified individualized targets for iTBS in each patient - the region of left DLPFC most anticorrelated with SCC activity. Intent-to-treat analysis revealed 86.4% (*N* = 19) of patients met remission criteria, without negative cognitive effects. In the follow-up double-blind RCT SNT study (2022), patients with TRD were enrolled and randomly assigned to one of two groups: sham (*N* = 15) or iTBS stimulation (*N* = 14) (Cole et al., 2022). At baseline and 4 weeks following treatment, patients completed the Montgomery-Asberg Depression Scale (MADRS). Patients in the stimulation group experienced a 52% reduction in depression scores. Compared to conventional TMS, the discovery of iTBS and new accelerated protocols may result in substantial clinical responses in a shorter time frame.

## Structural and functional connectivity of neural networks in depression: Salience as a key switching mechanism

Diffusion-weighted imaging (DWI) is a structural MR technique that derives several important physical tissue properties within the brain. These imaging markers can be used to extract brain network profiles and regions important for TRD pathophysiology. Fractional anisotropy and mean diffusivity, two of the most frequently reported metrics, provide information on microstructural architecture and integrity of white matter. Both metrics are obtained using diffusion tensor imaging, the analytical method forming the basis of tractography (Basser and Pierpaoli, 1996). Another variant of diffusion-weighted imaging is diffusion kurtosis imaging which quantifies the non-Gaussian quality of water diffusion (Jensen et al., 2005). Diffusion imaging has been used extensively to investigate how structural connectivity differs in TRD patients. Peng and colleagues conducted a double-blind, randomized study aimed at ascertaining whether white matter abnormalities cause network dysfunction in TRD patients (Peng et al., 2012). This group enrolled 30 TRD patients and randomized subjects to sham or active TMS treatment. The investigators acquired diffusion imaging and found significant reductions in FA in the left middle frontal gyrus not observed in the sham group. Korgaonkar et al. (2014) analyzed 102 MDD patients and found that the anterior cingulate cortex (ACC) limbic white matter is a useful predictor of antidepressant treatment outcome (Korgaonkar et al., 2014). Thus, based on DWI, FA could provide a glimpse into which patients with depression might be responsive to treatment.

Measurement of gray and white matter thickness using voxel-based morphometry (VBM) as well as connectivity between brain regions, inferred as specific white matter tracts or networks, can be extracted using a combination of structural and diffusion-weighted imaging. For example, the default mode network (DMN), which is distributed over many cortical regions, and the frontoparietal central executive network (CEN), have been shown to be dysregulated in patients with depression (Liston et al., 2014). DLPFC is in fact a node within the CEN. However, tractography studies were inconclusive as to whether DLPFC modulates connectivity between the two networks. Liston et al. (2014) sought to address this knowledge gap utilizing resting-state functional MRI (Liston et al., 2014). They enrolled 17 patients with depression and 35 healthy controls. The patients received 25 sessions of TMS over a 5 week period. Clinical scales assessing depression were completed at baseline and 1–3 days after completing the treatment period. Structural and diffusion-weighted scans were acquired before and after treatment. Functional connectivity maps were generated between the CEN, and DMN, using DLPFC as a seeding region. The authors observed that TMS of the DLPFC leads to connectivity changes in the DMN. This supports the idea that structures remote from the DLPFC such as DMN are also affected by TMS, suggesting a model based on knowledge of structural connectomics. However, very few studies have combined tractography and connectivity analyses in the same cohort with DWI and fMRI to determine the correlation between the two methods, particularly with respect to DLPFC and TMS.

The salience network (SN) is also dysfunctional in TRD, though it appears to have certain predictive characteristics for TMS response not seen in DMN or CEN. Fan et al. (2019) enrolled subjects with MDD and twenty healthy controls (Fan et al., 2019). Twenty real and sham sessions of TMS were administered to the DLPFC 5 days per week. Resting state fMRI sessions were performed before and after the TMS sessions, while clinical scales measuring depression were administered weekly. Segregation analyses were performed to index connections between and within networks. Using these analyses, the authors observed that segregation of the SN predicts symptom improvement after TMS, which adds to our understanding of the pathophysiology of depressive symptoms. Several other authors have hypothesized that SN performs a switching mechanism during effective treatment of depression, since it appears to refocus maladaptive ruminative behavior driven by DMN towards purposeful executive function and planning behavior driven by CEN (Sridharan et al., 2008; Menon, 2011; Goulden et al., 2014; Anderson et al., 2016). Tables 1, 2 summarize the changes in DMN, CEN, and SN associated with TRD.

**TABLE 1:** Intrinsic connectivity networks in treatment-resistant depression (TRD).

Network	Component brain regions	Function	Connectivity in TRD	References
Default mode network	VMPFC, DMPFC, PCC, inferior parietal lobe, hippocampal formation	● Decreased activity in goal-directed tasks ● Increased activity during self-referential processing and resting state	● Hyperconnectivity within DMN and between DMN and thalamus ● Hypoconnectivity between DMN and bilateral caudate	Greicius et al., 2007; Buckner et al., 2008; Andrews-Hanna et al., 2010; Liston et al., 2014; Kaiser et al., 2015; Anderson et al., 2016
Central executive network	DLPFC, lateral posterior parietal cortex, ventrolateral prefrontal cortex, thalamus	● Increased activity during goal-directed tasks requiring sustained attention and working memory	● Hypoconnectivity or Hyperconnectivity within the CEN	Fox et al., 2005; Seeley et al., 2007; Alexopoulos et al., 2012; Liston et al., 2014; Kaiser et al., 2015; Anderson et al., 2016
Salience network	dACC, frontoinsular cortex, amygdala, VTA	● Detection of personally salient and rewarding stimuli ● Integration of external and internal emotional, homeostatic, and cognitive nature ● Guiding of appropriate behavioral responses	● Hypoconnectivity within the SN relative to symptom severity ● Overactivity of dACC, insula, and amygdala when presented with stimuli of negative affect	Seeley et al., 2007; Menon, 2011; Hamilton et al., 2013; Goulden et al., 2014; Manoliu et al., 2014; Uddin, 2015; Anderson et al., 2016

VMPFC, ventromedial prefrontal cortex; DMPFC, dorsomedial prefrontal cortex; PCC, posterior cingulate cortex; DLPFC, dorsolateral prefrontal cortex; dACC, dorsal anterior cingulate cortex; VTA, ventral tegmental area.

**TABLE 2:** Network connectivity changes in treatment-resistant depression (TRD) and resultant symptoms.

Network	Connectivity increase or decrease in TRD	Associated symptom	References
Default mode network	1. ↑ Connectivity within default mode network	1. Rumination	Sheline et al., 2010; Hamilton et al., 2015; Williams, 2016
Central executive network	1. ↓ DLPFC-parietal cortex 2. ↓ ACC-DLPFC	1. Inattention, false alarm errors 2. Cognitive dysfunction, latency	Qiu et al., 2011; Sylvester et al., 2012; Forster et al., 2015; Williams, 2016
Salience network	1. ↑ Insula-amygdala 2. ↓ Insula-ACC 3. ↓ Amygdala-subcallosal and ventral ACC 4. Striatal hypoactivation 5. ACC hyperactivation	6. Anxious avoidance 7. Negative Bias 8. Threat dysregulation 9. Anhedonia 10. Context insensitivity	Matthews et al., 2008; Stuhrmann et al., 2011; Treadway and Zald, 2011; Klumpp et al., 2013; Zhang et al., 2013; Mulders et al., 2015; Williams et al., 2016

TRD, treatment resistant depression; ACC, anterior cingulate cortex; DLPFC, dorsolateral prefrontal cortex.

## Epidural cortical stimulation for treatment-resistant depression: Which is more effective – unilateral or bilateral stimulation of DLPFC?

Given the success and US FDA approval of TMS for MDD in 2008, epidural cortical stimulation (EpCS) followed as an expansion of neuromodulation tools explored for the treatment of TRD. In an open-label study of EpCS as an adjunctive therapy for TRD, 5 patients were enrolled who had previously failed electroconvulsive therapy (ECT), TMS, and vagus nerve stimulation (VNS). Each patient underwent implantation of bilateral paddle leads positioned epidurally over Brodmann’s areas 10 (frontopolar prefrontal cortex) and 46 (dorsolateral prefrontal cortex) (Nahas et al., 2010). After 7 months of follow-up, patients experienced significant improvement in depressive symptoms, compared to the pre-implantation baseline—with over 50% mean improvement in metrics of depression severity and 80% of the cohort achieving remission (Nahas et al., 2010; Williams et al., 2016, 2018). Remission was sustained at the 3 and 5 year follow-up timepoints, highlighting the potential of EpCS as an adjunctive therapy for TRD, following the failure of other modalities of neuromodulation.

Similar to TMS, EpCS generates peak electric fields at the gyral crown, subjacent to the stimulation site (Wongsarnpigoon and Grill, 2008). This suggests that the antidepressive effects may depend on direct activation of gray matter, recruitment of deeper white matter and subcortical structures or both. In a meta-analysis of resting state functional imaging studies, patients with depression displayed hypoactivation of the pregenual cingulate cortex (pgCC), dorsal anterior cingulate cortex (dACC), and insula (Fitzgerald et al., 2008). Interestingly, hypoactivity was significant for bilateral dorsolateral prefrontal cortices (DLPFC) in that study (Fitzgerald et al., 2008). In addition, Salomons et al. targeted bilateral prefrontal cortices and observed changes in connectivity between SN, CEN, and DMN networks following 20 sessions of 10 Hz TMS (Salomons et al., 2014; Anderson et al., 2016). These findings support the Nahas and Williams trials which resulted in up to 80% sustained remission (Nahas et al., 2010; Williams et al., 2016, 2018) while, in a separate study, unilateral (left-sided) conventional DLPFC stimulation produced an average of 30% remission (Kopell et al., 2011). This also highlights the potential to utilize EpCS as an alternative approach in TRD patients non-responsive to TMS, perhaps drawing on similar key mechanisms (Fitzgerald et al., 2008; Grimm et al., 2008).

In addition to direct modulation of hypoactivated DLPFC, EpCS of DLPFC could improve symptoms of TRD by altering connectivity with the subcallosal cingulate cortex (SCC), similar to TMS (Fox et al., 2012) or by indirectly modulating SN. As mentioned, the SN is hypothesized to act as a “switch” that serves to transition between other core functional networks (including CEN and DMN) recruited during emotion regulation and social cognition. Nodes in the SN include dACC and bilateral insular cortices. These regions have been designated “common core” regions with aberrant activation in many psychiatric disorders, including obsessive-compulsive and post-traumatic stress disorders (Downar et al., 2016). These relationships suggest a functional interaction between DLPFC and the SN that is possibly exploited via TMS (Table 3) and EpCS for the modulation of TRD. DLPFC may act as a superficial access point to drive modulation of the deeper salience network, functioning to improve mood regulation by normalizing the activation balance and promoting transitions between intrinsic and task-evoked circuits.

**TABLE 3:** Transcranial magnetic stimulation (TMS) studies focusing on salience network.

References	Study type	Modality	Sample	Region or network of interest	Key findings
Wada et al., 2022	N/A	TMS-EEG and MRI	60 patients with TRD and 30 healthy controls	DLPFC, SN	In patients with TRD, signal transmission from the left DLPFC to the salience network was reduced in the θ and α bands.
Iwabuchi et al., 2019	Randomized study	TMS and fMRI	27 patients with TRD	DLPFC, SN, Fronto-insular	Early response to rTMS in TRD can be predicted by fronto-insular and salience-network connections.
Hawco et al., 2018	N/A	TMS-fMRI	26 healthy young adults	DLPFC, SN	Changes induced by TMS following stimulation of the DLPFC are associated with resting state connectivity, particularly when the DLPFC target is engaged with SN.
Philip et al., 2018	Prospective trial	TMS and fMRI	33 adults receiving care at neuromodulation clinics at Brown University-affiliated hospitals.	SCC, DLPFC, SN	After TMS, symptom reduction was associated with reduced connectivity between the SCC and the default mode network, left dorsolateral prefrontal cortex, and insula, and reduced connectivity between the hippocampus and the salience network.
Schluter et al., 2018	Randomized, single-blind trial	HF-TMS	45 healthy controls	LFPN, RFPN, DMN, SN, and RN	The salience network had less functional connection when the left DLPFC was stimulated, but this network had more functional connectivity when the right DLPFC was stimulated.

MDD, major depression disorder; DMN, default mode network; SN, salience network; FPN, fronto-parietal network; CON, cingulo-opercular network; MRN, memory retrieval network; pDMN, posterior default mode network; SCC, subcallosal cingulate cortex; PCC, posterior cingulate cortex; FPN, fronto-parietal network; HF-TMS, high-frequency TMS; LFPN, left frontoparietal network; RFPN, right frontoparietal network; RN, reward network.

## Deep brain stimulation for treatment-resistant depression: Novel ideas emerge from clinical trials

Deep brain stimulation (DBS) is an invasive modality applied via stereotactic insertion of one or more intracranial leads. Its initial utility was investigated in the context of movement disorders, inspired by evidence that high frequency stimulation (100–200 Hz) of the ventral intermediate thalamic nucleus could alleviate tremor (Albe Fessard et al., 1963). In 1996, thalamic DBS received FDA approval in the US for essential and Parkinsonian tremors. Approvals for subthalamic nucleus (STN) and globus pallidus internus (GPi) DBS followed in 2003 for Parkinson’s Disease (Miocinovic et al., 2013) and anterior thalamic nucleus (ATN) DBS for epilepsy in 2018 (Salanova et al., 2015; Kim et al., 2017). Evidence-based application of DBS for TRD has proven complex given the promising outcomes in open-label investigations that have not been reproduced in randomized clinical trials (RCT) (Morishita et al., 2014). In cohort studies of nucleus accumbens DBS, 40–45% of the TRD cohort responded with > 50% reduction in depressive symptoms. For ventral capsule/ventral striatum (VC/VS) DBS cohort studies, response rates were 53% at 12 months, and 71% by the long-term endpoint (mean of 37 months) (Malone et al., 2009; Malone, 2010). Despite these positive results, the multicenter RCT of VC/VS DBS failed to meet its primary endpoint (Dougherty et al., 2015). Similarly, positive effects of subcallosal cingulate cortex (SCC) DBS were reported in multiple case reports (Neimat et al., 2008; Guinjoan et al., 2010; Holtzheimer and Mayberg, 2010; Hamani et al., 2012) and cohort studies demonstrating > 60% responder rate (Mayberg et al., 2005; Kennedy et al., 2011). A subsequent multicenter trial showed a 29% responder rate at 12 months (Lozano et al., 2012), while a single-blinded study by Holtzheimer et al. reported 91% response rate and 58% remission rate (Holtzheimer et al., 2012). Of note the later study utilized higher intensity stimulation (6–10 mA) than the former (5.2 mA). These led to the BROADEN trial, which was conducted as a multicenter, randomized controlled trial for SCC DBS. Subjects underwent bilateral SCC implantation and were randomized to active or sham DBS for 6 months. Following the first endpoint of 6 months, no group differences were found, and the study was discontinued following a futility analysis. Nevertheless, two novel findings emerged from this study. First, a retrospective tractography study observed that SCC DBS responders were more likely to have the active contacts located at the convergence of four white matter pathways: forceps minor, uncinate fasciculus, cingulum bundle, and fronto-striatal fibers (Riva-Posse et al., 2014). Second, when subjects were followed longitudinally (2–8 years), the response and remission rates rose to 81 and 54%, respectively (Crowell et al., 2019). These follow-up studies provide encouragement for the therapeutic potential of DBS for TRD, as increasing evidence suggests that heterogeneity of psychiatric disorders may warrant an individualized approach, including connectomics and tractography, for patient-specific target identification (Riva-Posse et al., 2018; Allawala et al., 2021; Hollunder et al., 2022).

### DBS antidepressive mechanisms

An advantage of SCC DBS is that it directly targets the white matter tracts underlying the subgenual cingulate cortex and thus gains access to functional networks involving the cingulum bundle. In contrast, the stimulus area accessed by ECT, TMS and EpCS is less focused. Notwithstanding, Tsolaki and colleagues demonstrated differences in responsivity to ECT based on SCC connectivity, again supporting the idea that non-invasive and invasive modalities share network coupling dynamics (Tsolaki et al., 2021). DBS contacts apply a three-dimensional electric field to surrounding tissue, resulting in depolarization or hyperpolarization of neighboring dendrites and axons (McIntyre and Foutz, 2013). The therapeutic effects are generated via high frequency stimulation (∼100–130 Hz) of a target selected for its ability to modulate an aspect of neurologic dysfunction such as ATN for epilepsy and STN for Parkinson’s disease. Initial functional studies in patients with depression demonstrated increased glucose metabolism in the SCC and pregenual cingulate cortex (pgCC) (Sacher et al., 2012). Mayberg and colleagues observed that hypermetabolism in SCC was attenuated with pharmacologic antidepressants (Mayberg et al., 1997, 2000), identifying SCC as a potential target for modulation of TRD with DBS. As suggested by tractography studies (Riva-Posse et al., 2014; Tsolaki et al., 2017), SCC DBS likely activates white matter tracts in close proximity to the electrode contacts, such as the cingulum bundle and uncinate fasciculus. The cingulum is a large white matter tract superficial to the corpus callosum and involved in executive control, emotion, pain, and memory (Bubb et al., 2018). Axons from the subgenual and anterior cingulate subsections have terminations in the anterior cingulate cortex (ACC), prefrontal cortex (dorsolateral, medial and orbital), amygdala, insula and superior temporal cortex (Bubb et al., 2018). Each of these structures have been strongly implicated in the SN and serve as potential sites of modulation for emotion dysregulation. Moreover, in a study of error rate in an emotional empathy task after ischemic stroke, Oishi et al. showed that right uncinate fasciculus lesions were associated with greater error rate. This demonstrated the role of the uncinate fasciculus, a tract with connections to orbitofrontal cortex, anterior insula, temporal pole, and amygdala, in emotional empathy (Oishi et al., 2015). SCC DBS at the intersection of these and other tracts may provide a locus for modulation of the SN given apparent overlap with key nodes: dorsal anterior cingulate cortex, anterior insula, temporal pole, and the amygdala (Friston, 2017). Direct access to the SN may provide a useful clue as to the potential mechanism for DMN and CEN activation via SCC DBS. Table 4 summarizes key clinical evidence of SN modulation with DBS.

**TABLE 4:** Deep brain stimulation (DBS) for treatment-resistant depression (TRD) focusing on salience network.

References	Study type	Modality	Sample	Region or network of interest	Key findings
Yan et al., 2021	Systematic review	DBS	4 cases of self-injurious behavior	SN, NAcc, ALIC	Abnormal pain processing is related to alterations of salience network connectivity which in turn may influence SIB.
Riva-Posse et al., 2019	Randomized trial	DBS	9 patients with TRD	SN, SCC	Salience of behavioral responses are associated with SCC DBS-induced autonomic changes.
Ellard et al., 2018	N/A	DBS	35 unipolar depressed patients, 24 bipolar depressed patients, and 39 healthy controls	SN, DMN, ECN	Patients with bipolar disease displayed weaker functional connectivity between right dorsal AI and right VLPFC (SN). Greater impairment in perceived control in unipolar depression correlated with stronger right dorsal AI – right VLPFC (SN) functional connectivity.
Downar et al., 2016	Review	DBS	N/A	SN, dACC, anterior insula	Behavioral self-control, emotion regulation, and social cognition show functional correlations with SN activity. aCIN, part of the SN, may be a potential neuropsychiatric DBS target.
Choi et al., 2015	Case series	DBS	9 TRD patients undergoing DBS	SN, SCC	Proximity to bilateral VMFC (via forceps minor and left uncinate fasciculus) and CC (via left cingulum bundle) correlate with higher structural connectivity and clinical response

Acin, anterior cingulo-insular; NAcc, nucleus accumbens; ALIC, anterior limb of the internal capsule; SIB, self-injurious behavior; SCC, subcallosal cingulate cortex; VMFC, ventromedial frontal cortex; CC, cingulate cortex; ECN, fronto-parietal executive control; AI, anterior insula; SCC, subcallosal cingulate.

## Multi-modality neuromodulation for TRD: A network-centric clinical algorithm

Given the complexity and heterogeneity associated with TRD, it would be advantageous to create a systematic therapeutic approach. We propose an heuristic clinical pathway that utilizes available neuromodulation technologies, tiered by invasiveness, to find the optimal, personalized treatment plan for individuals with TRD. This stepwise neuromodulation clinical pathway, paired with individualized target identification via tractography and functional connectomics, could result in more consistent patient outcomes.

**FIGURE 1:**
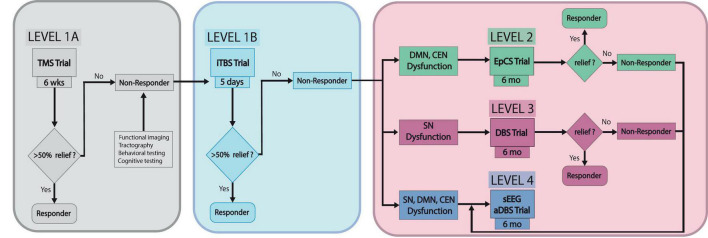
TRD neuromodulation clinical pathway. This schematic depicts the proposed stepwise pathway for optimizing individualized management of treatment-resistant depression sEEG, stereotactic electroencephalography; aDBS, adaptive deep brain stimulation.

In this pathway, patients that have failed three or more antidepressants and ECT will be treated with *Level 1A* neuromodulation—conventional DLPFC TMS protocol (6 weeks). If symptoms persist (<50% improvement in symptoms), patients may undergo additional testing including functional connectivity imaging, tractography, behavioral and cognitive testing for characterization of patient-specific TRD neurobiology contributing to depressive symptoms. Unique TRD profiles in certain patients have been suggested by several authors proposing that specific depressive symptoms map onto distinct functional networks (Williams, 2016; Hollunder et al., 2022). Imaging data would be used to identify personalized targets for subsequent neuromodulation. *The next step, Level 1B* neuromodulation, consists of accelerated TMS such as the 5 day SAINT protocol, using iTBS. If this fails, evidence of abnormal activation in DMN (hyperactivation) or CEN (hypoactivation) would be an indicator for *Level 2* neuromodulation—bilateral DLPFC and frontopolar PFC EpCS for 6 months. If imaging identifies hypoactivation of SN, *Level 3* neuromodulation—SCC DBS, would be recommended. VNS, which has been implicated in DLPFC activation via connections to the locus coeruleus, is an alternative if intracranial surgery is contraindicated (Conway et al., 2006, 2018; Dorr and Debonnel, 2006; Roosevelt et al., 2006; Kamel et al., 2022). Broader network dysfunction in DMN, CEN, and SN or severe, refractory cases would be an indication for *Level 4* neuromodulation—stereotactic electroencephalography (sEEG)-informed adaptive DBS. For this level, sEEG would assist with identifying symptom-specific biomarkers and targets for personalized closed-loop DBS. Scangos and colleagues used this approach and reported favorable outcomes with VC/VS stimulation for modulation of symptom-associated gamma power in the amygdala of one patient with TRD (Scangos et al., 2021). In that study, machine learning (ML) algorithms were used to analyze biomarkers in one structure while stimulating in a remote site. While specific white matter pathways were not considered in that study, it is interesting that the ML algorithms modeling the data identified a target other than SCC as most effective in ameliorating the patient’s symptoms. The findings support the idea that not only may TRD manifest differently in each individual, structures outside the traditional DMN, SN or CEN networks, such as the amygdala, may provide useful biomarker correlations. A schematic of the proposed clinical pathway is depicted in Figure 1.

As previously described, depression is associated with hyperactivation of the DMN which may contribute to symptoms of rumination and pessimism (Anderson et al., 2016). This may or may not be cosynchronous with SN and CEN hypoactivation, resulting in aberrant responses to salient stimuli, memory deficits, and attentional dysfunction (Anderson et al., 2016). The neuromodulation clinical pathway we have proposed enables a multifaceted approach for treating depression by targeting nodes within each of these networks. This provides a systematic approach for implementing neuromodulation to find the target and therapy that works optimally for each patient.

**FIGURE 2:**
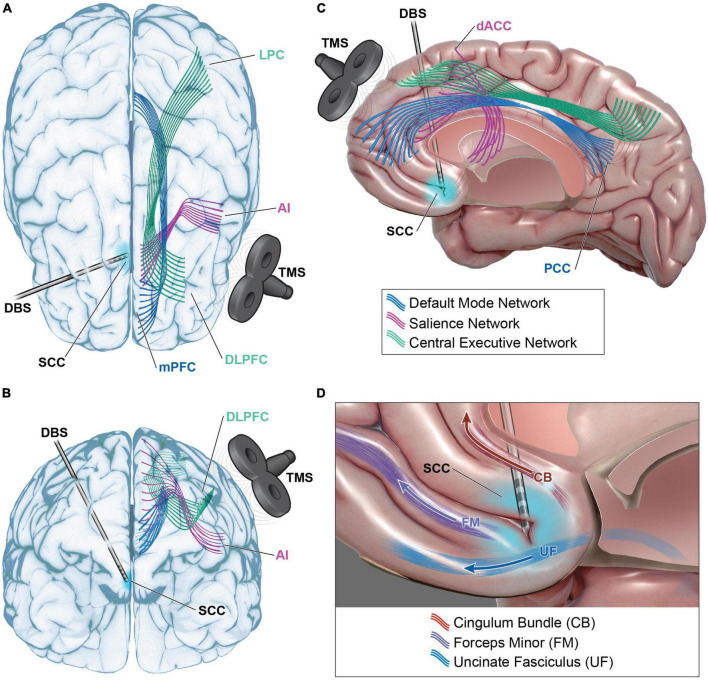
Functional network modulation in TRD. Default mode network (DMN – blue), salience network (SN – purple), and central executive network (CEN – green) are accessible for modulation with transcranial magnetic stimulation (TMS) and deep brain stimulation (DBS). Dorsal lateral prefrontal cortex (DLPFC) TMS may modulate CEN via direct modulation of DLPFC and its projections to lateral parietal cortex (LPC) **(A–C)**. DLPFC TMS could indirectly modulate SN via functional connections with anterior cingulate cortex (ACC), anterior insula (AI), or DMN via functional connections with medial prefrontal cortex (mPFC) and posterior cingulate cortex (PCC). DBS at the intersection of subcallosal cingulate cortex (SCC), the cingulum bundle (CB), uncinate fasciculus (UF), and forceps minor (FM) may modulate SN via projections to dorsal anterior cingulate cortex (dACC) and AI **(C,D)**. Functional connections to DLPFC, mPFC, and PCC also present avenues for modulation of CEN and DMN. Please see Table 1 for full listing of network structures.

In summary, the imbalance between network activity may prime individuals to preferentially respond to a particular stimulation locus and modality. TMS at the DLPFC directly modulates the node of the central executive network. But this region also has functional connections with nodes of the SN (ACC and anterior insula) and the DMN (ventromedial prefrontal cortex (VMPFC), posterior cingulate cortex (PCC) and lateral parietal cortex) (Figures 2A–C). Patients with pathologic hypoactivation of DLPFC as the primary insult may be responders to TMS. Bilateral DLPFC and frontopolar stimulation with EpCS likely alters activity of the CEN and DMN but also has downstream effects on the salience network. DBS at the intersection of the SCC, cingulum, and uncinate fasciculus likely modulates the SN via dACC and anterior insula (Figures 2C, D). This is probably paired with effects on the CEN and DMN through functional connections with DLPFC and VMPFC to PCC, respectively. Patients with hypoactivation of the salience network may preferentially respond to SCC DBS. A network-centric clinical pathway brings together multiple disciplines and proposes a new common language with the aim of enhancing care for treatment-resistant depression.

## Author contributions

NR and ES helped to frame to scope of the manuscript and provided the editorial support. SI-A, CS, MB, and NR composed the manuscript, figures, and tables. All authors contributed to the article and approved the submitted version.

## References

[B1] Albe Fessard D. Arfel G. Guiot G. Derome P. Dela H. Korn H. (1963). Characteristic electric activities of some cerebral structures in man. *Ann. Chir.* 17 1185–1214. 14084565

[B2] Alexopoulos G. Hoptman M. Kanellopoulos D. Murphy C. Lim K. Gunning F. (2012). Functional connectivity in the cognitive control network and the default mode network in late-life depression. *J. Affect. Disord.* 139 56–65. 10.1016/j.jad.2011.12.002 22425432 PMC3340472

[B3] Al-Harbi K. (2012). Treatment-resistant depression: therapeutic trends, challenges, and future directions. *Patient Prefer Adher.* 6 369–388. 10.2147/PPA.S29716 22654508 PMC3363299

[B4] Allawala A. Bijanki K. Goodman W. Cohn J. Viswanathan A. Yoshor D. (2021). A novel framework for network-targeted neuropsychiatric deep brain stimulation. *Neurosurgery* 89 E116–E121. 10.1093/neuros/nyab112 33913499 PMC8279838

[B5] Anderson R. Hoy K. Daskalakis Z. Fitzgerald P. (2016). Repetitive transcranial magnetic stimulation for treatment resistant depression: Re-establishing connections. *Clin. Neurophysiol.* 127 3394–3405. 10.1016/j.clinph.2016.08.015 27672727

[B6] Andrews-Hanna J. Reidler J. Sepulcre J. Poulin R. Buckner R. (2010). Functional-anatomic fractionation of the brain’s default network. *Neuron* 65 550–562. 10.1016/j.neuron.2010.02.005 20188659 PMC2848443

[B7] Barker A. Jalinous R. Freeston I. (1985). Non-invasive magnetic stimulation of human motor cortex. *Lancet* 1 1106–1107. 10.1016/s0140-6736(85)92413-4 2860322

[B8] Basser P. Pierpaoli C. (1996). Microstructural and physiological features of tissues elucidated by quantitative-diffusion-tensor MRI. *J. Magn. Reson. B.* 111 209–219. 10.1006/jmrb.1996.0086 8661285

[B9] Bergfeld I. Mantione M. Figee M. Schuurman P. Lok A. Denys D. (2018). Treatment-resistant depression and suicidality. *J. Affect. Disord.* 235 362–367. 10.1016/j.jad.2018.04.016 29665520

[B10] Berman R. Narasimhan M. Sanacora G. Miano A. Hoffman R. Hu X. (2000). A randomized clinical trial of repetitive transcranial magnetic stimulation in the treatment of major depression. *Biol. Psychiatry* 47 332–337. 10.1016/s0006-3223(99)00243-7 10686268

[B11] Blumberger D. Vila-Rodriguez F. Thorpe K. Feffer K. Noda Y. Giacobbe P. (2018). Effectiveness of theta burst versus high-frequency repetitive transcranial magnetic stimulation in patients with depression (THREE-D): A randomised non-inferiority trial. *Lancet* 391 1683–1692. 10.1016/S0140-6736(18)30295-2 29726344

[B12] Bubb E. Metzler-Baddeley C. Aggleton J. (2018). The cingulum bundle: Anatomy, function, and dysfunction. *Neurosci. Biobehav. Rev.* 92 104–127. 10.1016/j.neubiorev.2018.05.008 29753752 PMC6090091

[B13] Buckner R. Andrews-Hanna J. Schacter D. (2008). The brain’s default network: Anatomy, function, and relevance to disease. *Ann. N. Y. Acad. Sci.* 1124 1–38. 10.1196/annals.1440.011 18400922

[B14] Choi K. Riva-Posse P. Gross R. Mayberg H. (2015). Mapping the “Depression Switch” During Intraoperative Testing of Subcallosal Cingulate Deep Brain Stimulation. *JAMA Neurol.* 72 1252–1260. 10.1001/jamaneurol.2015.2564 26408865 PMC4834289

[B15] Cohen S. Bikson M. Badran B. George M. S. (2022). A visual and narrative timeline of US FDA milestones for Transcranial Magnetic Stimulation (TMS) devices. *Brain Stimul.* 15 73–75. 10.1016/j.brs.2021.11.010 34775141 PMC8864803

[B16] Cole E. Phillips A. Bentzley B. Stimpson K. Nejad R. Barmak F. (2022). Stanford Neuromodulation Therapy (SNT): A double-blind randomized controlled trial. *Am. J. Psychiatry* 179 132–141. 10.1176/appi.ajp.2021.20101429 34711062

[B17] Cole E. Stimpson K. Bentzley B. Gulser M. Cherian K. Tischler C. (2020). Stanford accelerated intelligent neuromodulation therapy for treatment-resistant depression. *Am. J. Psychiatry* 177 716–726. 10.1176/appi.ajp.2019.19070720 32252538

[B18] Conway C. Kumar A. Xiong W. Bunker M. Aaronson S. Rush A. (2018). Chronic vagus nerve stimulation significantly improves quality of life in treatment-resistant major depression. *J. Clin. Psychiatry* 79:18m12178. 10.4088/JCP.18m12178 30152645

[B19] Conway C. Sheline Y. Chibnall J. George M. Fletcher J. Mintun M. (2006). Cerebral blood flow changes during vagus nerve stimulation for depression. *Psychiatry Res.* 146 179–184. 10.1016/j.pscychresns.2005.12.007 16510266

[B20] Crowell A. Riva-Posse P. Holtzheimer P. Garlow S. Kelley M. Gross R. (2019). Long-Term outcomes of subcallosal cingulate deep brain stimulation for treatment-resistant depression. *Am. J. Psychiatry* 176 949–956. 10.1176/appi.ajp.2019.18121427 31581800

[B21] Dorr A. Debonnel G. (2006). Effect of vagus nerve stimulation on serotonergic and noradrenergic transmission. *J. Pharmacol. Exp. Ther.* 318 890–898. 10.1124/jpet.106.104166 16690723

[B22] Dougherty D. Rezai A. Carpenter L. Howland R. Bhati M. O’Reardon J. (2015). A Randomized Sham-Controlled Trial of Deep Brain Stimulation of the Ventral Capsule/Ventral Striatum for Chronic Treatment-Resistant Depression. *Biol. Psychiatry* 78 240–248. 10.1016/j.biopsych.2014.11.023 25726497

[B23] Downar J. Blumberger D. Daskalakis Z. (2016). The neural crossroads of psychiatric illness: An emerging target for brain stimulation. *Trends Cogn. Sci.* 20 107–120. 10.1016/j.tics.2015.10.007 26655436

[B24] Ellard K. Zimmerman J. Kaur N. Van Dijk K. Roffman J. Nierenberg A. (2018). Functional connectivity between anterior insula and key nodes of frontoparietal executive control and salience networks distinguish bipolar depression from unipolar depression and healthy control subjects. *Biol. Psychiatry Cogn. Neurosci. Neuroimaging* 3 473–484. 10.1016/j.bpsc.2018.01.013 29580768 PMC6467478

[B25] Fan J. Tso I. Maixner D. Abagis T. Hernandez-Garcia L. Taylor S. (2019). Segregation of salience network predicts treatment response of depression to repetitive transcranial magnetic stimulation. *Neuroimage Clin.* 22:101719. 10.1016/j.nicl.2019.101719 30776777 PMC6378906

[B26] Fitzgerald P. Laird A. Maller J. Daskalakis Z. J. (2008). A meta-analytic study of changes in brain activation in depression. *Hum. Brain Mapp.* 29 683–695. 10.1002/hbm.20426 17598168 PMC2873772

[B27] Forster S. Nunez Elizalde A. Castle E. Bishop S. (2015). Unraveling the anxious mind: Anxiety, worry, and frontal engagement in sustained attention versus off-task processing. *Cereb. Cortex* 25 609–618. 10.1093/cercor/bht248 24062316 PMC4318530

[B28] Fox M. Buckner R. White M. Greicius M. Pascual-Leone A. (2012). Efficacy of transcranial magnetic stimulation targets for depression is related to intrinsic functional connectivity with the subgenual cingulate. *Biol. Psychiatry* 72 595–603. 10.1016/j.biopsych.2012.04.028 22658708 PMC4120275

[B29] Fox M. Snyder A. Vincent J. Corbetta M. Van Essen D. Raichle M. (2005). The human brain is intrinsically organized into dynamic, anticorrelated functional networks. *Proc. Natl. Acad. Sci. U.S.A.* 102 9673–9678. 10.1073/pnas.0504136102 15976020 PMC1157105

[B30] Friston K. (2017). Precision Psychiatry. *Biol. Psychiatry Cogn. Neurosci. Neuroimaging* 2 640–643. 10.1016/j.bpsc.2017.08.007 29560899

[B31] George M. Lisanby S. Avery D. McDonald W. Durkalski V. Pavlicova M. (2010). Daily left prefrontal transcranial magnetic stimulation therapy for major depressive disorder: A sham-controlled randomized trial. *Arch. Gen. Psychiatry* 67 507–516. 10.1001/archgenpsychiatry.2010.46 20439832

[B32] George M. Nahas Z. Molloy M. Speer A. Oliver N. Li X. (2000). A controlled trial of daily left prefrontal cortex TMS for treating depression. *Biol. Psychiatry* 48 962–970. 10.1016/s0006-3223(00)01048-9 11082469

[B33] Goulden N. Khusnulina A. Davis N. Bracewell R. Bokde A. McNulty J. (2014). The salience network is responsible for switching between the default mode network and the central executive network: Replication from DCM. *Neuroimage* 99 180–190. 10.1016/j.neuroimage.2014.05.052 24862074

[B34] Greicius M. Flores B. Menon V. Glover G. Solvason H. Kenna H. (2007). Resting-state functional connectivity in major depression: Abnormally increased contributions from subgenual cingulate cortex and thalamus. *Biol. Psychiatry* 62 429–437. 10.1016/j.biopsych.2006.09.020 17210143 PMC2001244

[B35] Grimm S. Beck J. Schuepbach D. Hell D. Boesiger P. Bermpohl F. (2008). Imbalance between left and right dorsolateral prefrontal cortex in major depression is linked to negative emotional judgment: An fMRI study in severe major depressive disorder. *Biol. Psychiatry* 63 369–376. 10.1016/j.biopsych.2007.05.033 17888408

[B36] Guinjoan S. Mayberg H. Costanzo E. Fahrer R. Tenca E. Antico J. (2010). Asymmetrical contribution of brain structures to treatment-resistant depression as illustrated by effects of right subgenual cingulum stimulation. *J. Neuropsychiatry Clin. Neurosci.* 22 265–277. 10.1176/jnp.2010.22.3.265 20686133

[B37] Hamani C. Giacobbe P. Diwan M. Balbino E. Tong J. Bridgman A. (2012). Monoamine oxidase inhibitors potentiate the effects of deep brain stimulation. *Am. J. Psychiatry* 169 1320–1321. 10.1176/appi.ajp.2012.12060754 23212066 PMC5756069

[B38] Hamilton J. Chen M. Gotlib I. (2013). Neural systems approaches to understanding major depressive disorder: An intrinsic functional organization perspective. *Neurobiol. Dis.* 52 4–11. 10.1016/j.nbd.2012.01.015 23477309 PMC3596788

[B39] Hamilton J. Farmer M. Fogelman P. Gotlib I. (2015). Depressive rumination, the default-mode network, and the dark matter of clinical neuroscience. *Biol. Psychiatry* 78 224–230. 10.1016/j.biopsych.2015.02.020 25861700 PMC4524294

[B40] Hawco C. Voineskos A. Steeves J. Dickie E. Viviano J. Downar J. (2018). Spread of activity following TMS is related to intrinsic resting connectivity to the salience network: A concurrent TMS-fMRI study. *Cortex* 108 160–172. 10.1016/j.cortex.2018.07.010 30195825

[B41] Hollunder B. Rajamani N. Siddiqi S. Finke C. Kühn A. Mayberg H. (2022). Toward personalized medicine in connectomic deep brain stimulation. *Prog. Neurobiol.* 210:102211. 10.1016/j.pneurobio.2021.102211 34958874

[B42] Holtzheimer P. Kelley M. Gross R. Filkowski M. Garlow S. Barrocas A. (2012). Subcallosal cingulate deep brain stimulation for treatment-resistant unipolar and bipolar depression. *Arch. Gen. Psychiatry* 69 150–158. 10.1001/archgenpsychiatry.2011.1456 22213770 PMC4423545

[B43] Holtzheimer P. Mayberg H. (2010). Deep brain stimulation for treatment-resistant depression. *Am. J. Psychiatry* 167 1437–1444. 10.1176/appi.ajp.2010.10010141 21131410 PMC4413473

[B44] Holtzheimer P. McDonald W. Mufti M. Kelley M. Quinn S. Corso G. (2010). Accelerated repetitive transcranial magnetic stimulation for treatment-resistant depression. *Depress Anxiety* 27 960–963. 10.1002/da.20731 20734360 PMC3020591

[B45] Iwabuchi S. Auer D. Lankappa S. Palaniyappan L. (2019). Baseline effective connectivity predicts response to repetitive transcranial magnetic stimulation in patients with treatment-resistant depression. *Eur. Neuropsychopharmacol.* 29 681–690. 10.1016/j.euroneuro.2019.02.012 30827757

[B46] Jensen J. Helpern J. Ramani A. Lu H. Kaczynski K. (2005). Diffusional kurtosis imaging: The quantification of non-gaussian water diffusion by means of magnetic resonance imaging. *Magn. Reson. Med.* 53 1432–1440. 10.1002/mrm.20508 15906300

[B47] Kaiser R. Andrews-Hanna J. Wager T. Pizzagalli D. (2015). Large-scale network dysfunction in major depressive disorder: A meta-analysis of resting-state functional connectivity. *JAMA Psychiatry* 72 603–611. 10.1001/jamapsychiatry.2015.0071 25785575 PMC4456260

[B48] Kamel L. Xiong W. Gott B. Kumar A. Conway C. (2022). Vagus nerve stimulation: An update on a novel treatment for treatment-resistant depression. *J. Neurol. Sci.* 434:120171. 10.1016/j.jns.2022.120171 35158102

[B49] Kennedy S. Giacobbe P. Rizvi S. Placenza F. Nishikawa Y. Mayberg H. (2011). Deep brain stimulation for treatment-resistant depression: Follow-up after 3 to 6 years. *Am. J. Psychiatry* 168 502–510. 10.1176/appi.ajp.2010.10081187 21285143

[B50] Kim S. Lim S. Kim J. Son B. Lee K. Shon Y. (2017). Long-term follow-up of anterior thalamic deep brain stimulation in epilepsy: A 11-year, single center experience. *Seizure* 52 154–161. 10.1016/j.seizure.2017.10.009 29040867

[B51] Klumpp H. Post D. Angstadt M. Fitzgerald D. Phan K. (2013). Anterior cingulate cortex and insula response during indirect and direct processing of emotional faces in generalized social anxiety disorder. *Biol. Mood Anxiety Disord.* 3:7. 10.1186/2045-5380-3-7 23547713 PMC3632493

[B52] Kopell B. Halverson J. Butson C. Dickinson M. Bobholz J. Harsch H. (2011). Epidural cortical stimulation of the left dorsolateral prefrontal cortex for refractory major depressive disorder. *Neurosurgery* 69 1015–1029. 10.1227/NEU.0b013e318229cfcd 21709597

[B53] Korgaonkar M. Williams L. Song Y. Usherwood T. Grieve S. (2014). Diffusion tensor imaging predictors of treatment outcomes in major depressive disorder. *Br. J Psychiatry* 205 321–328. 10.1192/bjp.bp.113.140376 24970773

[B54] Lisanby S. Husain M. Rosenquist P. Maixner D. Gutierrez R. Krystal A. (2009). Daily left prefrontal repetitive transcranial magnetic stimulation in the acute treatment of major depression: Clinical predictors of outcome in a multisite, randomized controlled clinical trial. *Neuropsychopharmacology* 34 522–534. 10.1038/npp.2008.118 18704101

[B55] Liston C. Chen A. Zebley B. Drysdale A. Gordon R. Leuchter B. (2014). Default mode network mechanisms of transcranial magnetic stimulation in depression. *Biol. Psychiatry* 76 517–526. 10.1016/j.biopsych.2014.01.023 24629537 PMC4209727

[B56] Lozano A. Giacobbe P. Hamani C. Rizvi S. Kennedy S. Kolivakis T. (2012). A multicenter pilot study of subcallosal cingulate area deep brain stimulation for treatment-resistant depression. *J. Neurosurg.* 116 315–322. 10.3171/2011.10.JNS102122 22098195

[B57] Malone D. (2010). Use of deep brain stimulation in treatment-resistant depression. *Cleve. Clin J. Med.* 77 (Suppl. 3) S77–S80. 10.3949/ccjm.77.s3.14 20622083

[B58] Malone D. Dougherty D. Rezai A. Carpenter L. Friehs G. Eskandar E. (2009). Deep brain stimulation of the ventral capsule/ventral striatum for treatment-resistant depression. *Biol. Psychiatry* 65 267–275. 10.1016/j.biopsych.2008.08.029 18842257 PMC3486635

[B59] Manoliu A. Riedl V. Zherdin A. Mühlau M. Schwerthöffer D. Scherr M. (2014). Aberrant dependence of default mode/central executive network interactions on anterior insular salience network activity in schizophrenia. *Schizophr. Bull.* 40 428–437. 10.1093/schbul/sbt037 23519021 PMC3932085

[B60] Matthews S. Strigo I. Simmons A. Yang T. Paulus M. (2008). Decreased functional coupling of the amygdala and supragenual cingulate is related to increased depression in unmedicated individuals with current major depressive disorder. *J. Affect. Disord.* 111 13–20. 10.1016/j.jad.2008.05.022 18603301

[B61] Mayberg H. Brannan S. Mahurin R. Jerabek P. Brickman J. Tekell J. (1997). Cingulate function in depression: A potential predictor of treatment response. *Neuroreport* 8 1057–1061. 10.1097/00001756-199703030-00048 9141092

[B62] Mayberg H. Brannan S. Tekell J. Silva J. Mahurin R. McGinnis S. (2000). Regional metabolic effects of fluoxetine in major depression: Serial changes and relationship to clinical response. *Biol. Psychiatry* 48 830–843. 10.1016/s0006-3223(00)01036-2 11063978

[B63] Mayberg H. Lozano A. Voon V. McNeely H. Seminowicz D. Hamani C. (2005). Deep brain stimulation for treatment-resistant depression. *Neuron* 45 651–660. 10.1016/j.neuron.2005.02.014 15748841

[B64] McIntyre C. Foutz T. (2013). Computational modeling of deep brain stimulation. *Handb. Clin. Neurol.* 116 55–61. 10.1016/B978-0-444-53497-2.00005-X 24112884 PMC5570759

[B65] Mendlowitz A. Shanbour A. Downar J. Vila-Rodriguez F. Daskalakis Z. Isaranuwatchai W. (2019). Implementation of intermittent theta burst stimulation compared to conventional repetitive transcranial magnetic stimulation in patients with treatment resistant depression: A cost analysis. *PLoS One* 14:e0222546. 10.1371/journal.pone.0222546 31513675 PMC6742475

[B66] Menon V. (2011). Large-scale brain networks and psychopathology: A unifying triple network model. *Trends Cogn. Sci.* 15 483–506. 10.1016/j.tics.2011.08.003 21908230

[B67] Miocinovic S. Somayajula S. Chitnis S. Vitek J. (2013). History, applications, and mechanisms of deep brain stimulation. *JAMA Neurol.* 70 163–171. 10.1001/2013.jamaneurol.45 23407652

[B68] Morishita T. Fayad S. Higuchi M. Nestor K. Foote K. (2014). Deep brain stimulation for treatment-resistant depression: Systematic review of clinical outcomes. *Neurotherapeutics* 11 475–484. 10.1007/s13311-014-0282-1 24867326 PMC4121451

[B69] Mulders P. van Eijndhoven P. Schene A. Beckmann C. Tendolkar I. (2015). Resting-state functional connectivity in major depressive disorder: A review. *Neurosci. Biobehav. Rev.* 56 330–344. 10.1016/j.neubiorev.2015.07.014 26234819

[B70] Nahas Z. Anderson B. Borckardt J. Arana A. George M. Reeves S. (2010). Bilateral epidural prefrontal cortical stimulation for treatment-resistant depression. *Biol. Psychiatry* 67 101–109. 10.1016/j.biopsych.2009.08.021 19819427 PMC2863140

[B71] Neimat J. Hamani C. Giacobbe P. Merskey H. Kennedy S. Mayberg H. (2008). Neural stimulation successfully treats depression in patients with prior ablative cingulotomy. *Am. J. Psychiatry* 165 687–693. 10.1176/appi.ajp.2008.07081298 18519534

[B72] Oishi K. Faria A. Hsu J. Tippett D. Mori S. Hillis A. (2015). Critical role of the right uncinate fasciculus in emotional empathy. *Ann. Neurol.* 77 68–74. 10.1002/ana.24300 25377694 PMC4293256

[B73] O’Reardon J. Solvason H. Janicak P. Sampson S. Isenberg K. Nahas Z. (2010). Reply regarding “efficacy and safety of transcranial magnetic stimulation in the acute treatment of major depression: A multisite randomized controlled trial”. *Biol. Psychiatry* 67 e15–e17. 10.1016/j.biopsych.2009.06.027 19914602

[B74] Pascual-Leone A. Valls-Solé J. Wassermann E. Hallett M. (1994). Responses to rapid-rate transcranial magnetic stimulation of the human motor cortex. *Brain* 117 847–858. 10.1093/brain/117.4.847 7922470

[B75] Pashut T. Wolfus S. Friedman A. Lavidor M. Bar-Gad I. Yeshurun Y. (2011). Mechanisms of magnetic stimulation of central nervous system neurons. *PLoS Comput. Biol.* 7:e1002022. 10.1371/journal.pcbi.1002022 21455288 PMC3063755

[B76] Peng H. Zheng H. Li L. Liu J. Zhang Y. Shan B. (2012). High-frequency rTMS treatment increases white matter FA in the left middle frontal gyrus in young patients with treatment-resistant depression. *J. Affect. Disord.* 136 249–257. 10.1016/j.jad.2011.12.006 22217432

[B77] Philip N. Barredo J. van ’t Wout-Frank M. Tyrka A. Price L. Carpenter L. (2018). Network Mechanisms of Clinical Response to Transcranial Magnetic Stimulation in Posttraumatic Stress Disorder and Major Depressive Disorder. *Biol. Psychiatry* 83 263–272. 10.1016/j.biopsych.2017.07.021 28886760 PMC6679924

[B78] Qiu C. Liao W. Ding J. Feng Y. Zhu C. Nie X. (2011). Regional homogeneity changes in social anxiety disorder: A resting-state fMRI study. *Psychiatry Res.* 194 47–53. 10.1016/j.pscychresns.2011.01.010 21831605

[B79] Riva-Posse P. Choi K. Holtzheimer P. Crowell A. Garlow S. Rajendra J. (2018). A connectomic approach for subcallosal cingulate deep brain stimulation surgery: Prospective targeting in treatment-resistant depression. *Mol. Psychiatry* 23 843–849. 10.1038/mp.2017.59 28397839 PMC5636645

[B80] Riva-Posse P. Choi K. Holtzheimer P. McIntyre C. Gross R. Chaturvedi A. (2014). Defining critical white matter pathways mediating successful subcallosal cingulate deep brain stimulation for treatment-resistant depression. *Biol. Psychiatry* 76 963–969. 10.1016/j.biopsych.2014.03.029 24832866 PMC4487804

[B81] Riva-Posse P. Inman C. Choi K. Crowell A. Gross R. Hamann S. (2019). Autonomic arousal elicited by subcallosal cingulate stimulation is explained by white matter connectivity. *Brain Stimul.* 12 743–751. 10.1016/j.brs.2019.01.015 30738778

[B82] Roosevelt R. Smith D. Clough R. Jensen R. Browning R. (2006). Increased extracellular concentrations of norepinephrine in cortex and hippocampus following vagus nerve stimulation in the rat. *Brain Res.* 1119 124–132. 10.1016/j.brainres.2006.08.048 16962076 PMC1751174

[B83] Sacher J. Neumann J. Fünfstück T. Soliman A. Villringer A. Schroeter M. (2012). Mapping the depressed brain: A meta-analysis of structural and functional alterations in major depressive disorder. *J. Affect. Disord.* 140 142–148. 10.1016/j.jad.2011.08.001 21890211

[B84] Salanova V. Witt T. Worth R. Henry T. Gross R. Nazzaro J. (2015). Long-term efficacy and safety of thalamic stimulation for drug-resistant partial epilepsy. *Neurology* 84 1017–1025. 10.1212/WNL.0000000000001334 25663221 PMC4352097

[B85] Salomons T. Dunlop K. Kennedy S. Flint A. Geraci J. Giacobbe P. (2014). Resting-state cortico-thalamic-striatal connectivity predicts response to dorsomedial prefrontal rTMS in major depressive disorder. *Neuropsychopharmacology* 39 488–498. 10.1038/npp.2013.222 24150516 PMC3870791

[B86] Scangos K. Khambhati A. Daly P. Makhoul G. Sugrue L. Zamanian H. (2021). Closed-loop neuromodulation in an individual with treatment-resistant depression. *Nat. Med.* 27 1696–1700. 10.1038/s41591-021-01480-w 34608328 PMC11219029

[B87] Schluter R. Jansen J. van Holst R. van den Brink W. Goudriaan A. (2018). Differential Effects of Left and Right Prefrontal High-Frequency Repetitive Transcranial Magnetic Stimulation on Resting-State Functional Magnetic Resonance Imaging in Healthy Individuals. *Brain Connect.* 8 60–67. 10.1089/brain.2017.0542 29237276

[B88] Seeley W. Menon V. Schatzberg A. Keller J. Glover G. Kenna H. (2007). Dissociable intrinsic connectivity networks for salience processing and executive control. *J. Neurosci.* 27 2349–2356. 10.1523/JNEUROSCI.5587-06.2007 17329432 PMC2680293

[B89] Sheline Y. Price J. Yan Z. Mintun M. (2010). Resting-state functional MRI in depression unmasks increased connectivity between networks via the dorsal nexus. *Proc. Natl. Acad. Sci. U.S.A.* 107 11020–11025. 10.1073/pnas.1000446107 20534464 PMC2890754

[B90] Souery D. Papakostas G. I. Trivedi M. H. (2006). Treatment-Resistant Depression. *J. Clin. Psychiatry* 67 (Suppl. 6) 16–22. 16848672

[B91] Sridharan D. Levitin D. Menon V. (2008). A critical role for the right fronto-insular cortex in switching between central-executive and default-mode networks. *Proc. Natl. Acad. Sci. U.S.A.* 105 12569–12574. 10.1073/pnas.0800005105 18723676 PMC2527952

[B92] Stuhrmann A. Suslow T. Dannlowski U. (2011). Facial emotion processing in major depression: A systematic review of neuroimaging findings. *Biol. Mood Anxiety Disord.* 1:10. 10.1186/2045-5380-1-10 22738433 PMC3384264

[B93] Sylvester C. Corbetta M. Raichle M. Rodebaugh T. Schlaggar B. Sheline Y. (2012). Functional network dysfunction in anxiety and anxiety disorders. *Trends Neurosci.* 35 527–535. 10.1016/j.tins.2012.04.012 22658924 PMC3432139

[B94] Tofts P. (1990). The distribution of induced currents in magnetic stimulation of the nervous system. *Phys. Med Biol.* 35 1119–1128. 10.1088/0031-9155/35/8/008 2217537

[B95] Treadway M. Zald D. (2011). Reconsidering anhedonia in depression: Lessons from translational neuroscience. *Neurosci. Biobehav. Rev.* 35 537–555. 10.1016/j.neubiorev.2010.06.006 20603146 PMC3005986

[B96] Tsolaki E. Espinoza R. Pouratian N. (2017). Using probabilistic tractography to target the subcallosal cingulate cortex in patients with treatment resistant depression. *Psychiatry Res. Neuroimaging* 261 72–74. 10.1016/j.pscychresns.2017.01.006 28142056 PMC5552295

[B97] Tsolaki E. Narr K. Espinoza R. Wade B. Hellemann G. Kubicki A. (2021). Subcallosal Cingulate Structural Connectivity Differs in Responders and Nonresponders to Electroconvulsive Therapy. *Biol. Psychiatry Cogn. Neurosci. Neuroimaging* 6 10–19. 10.1016/j.bpsc.2020.05.010 32741703

[B98] Uddin L. (2015). Salience processing and insular cortical function and dysfunction. *Nat. Rev. Neurosci.* 16 55–61. 10.1038/nrn3857 25406711

[B99] Wada M. Nakajima S. Honda S. Takano M. Taniguchi K. Tsugawa S. (2022). Reduced signal propagation elicited by frontal transcranial magnetic stimulation is associated with oligodendrocyte abnormalities in treatment-resistant depression. *J. Psychiatry Neurosci.* 47 E325–E335. 10.1503/jpn.220102 36104082 PMC9484613

[B100] Williams L. M. (2016). Precision psychiatry: A neural circuit taxonomy for depression and anxiety. *Lancet Psychiatry* 3 472–480. 10.1016/S2215-0366(15)00579-9 27150382 PMC4922884

[B101] Williams N. Bentzley B. Hopkins T. Pannu J. Sahlem G. Takacs I. (2018). Optimization of epidural cortical stimulation for treatment-resistant depression. *Brain Stimul.* 11 239–240. 10.1016/j.brs.2017.09.001 28918944

[B102] Williams N. Short E. Hopkins T. Bentzley B. Sahlem G. Pannu J. (2016). Five-Year Follow-Up of Bilateral Epidural Prefrontal Cortical Stimulation for Treatment-Resistant Depression. *Brain Stimul.* 9 897–904. 10.1016/j.brs.2016.06.054 27443912

[B103] Williams N. Sudheimer K. Cole E. Varias A. Goldstein-Piekarski A. Stetz P. (2021). Accelerated neuromodulation therapy for Obsessive-Compulsive Disorder. *Brain Stimul.* 14 435–437. 10.1016/j.brs.2021.02.013 33631349 PMC8114181

[B104] Wongsarnpigoon A. Grill W. (2008). Computational modeling of epidural cortical stimulation. *J. Neural Eng.* 5 443–454. 10.1088/1741-2560/5/4/009 19015584

[B105] Yan H. Elkaim L. Loh A. Boutet A. Germann J. Elias G. (2021). Lesions causing self-injurious behavior engage putative networks modulated by deep brain stimulation. *Brain Stimul.* 14 273–276. 10.1016/j.brs.2021.01.009 33482373

[B106] Zhang W. Chang S. Guo L. Zhang K. Wang J. (2013). The neural correlates of reward-related processing in major depressive disorder: A meta-analysis of functional magnetic resonance imaging studies. *J. Affect. Disord.* 151 531–539. 10.1016/j.jad.2013.06.039 23856280

